# Comprehensive analysis of DNA damage repair genes reveals pathogenic variants beyond *BRCA* and suggests the need for extensive genetic testing in pancreatic cancer

**DOI:** 10.1186/s12885-021-08368-5

**Published:** 2021-05-26

**Authors:** Ilario Giovanni Rapposelli, Valentina Zampiga, Ilaria Cangini, Valentina Arcangeli, Mila Ravegnani, Martina Valgiusti, Sara Pini, Stefano Tamberi, Giulia Bartolini, Alessandro Passardi, Giovanni Martinelli, Daniele Calistri, Giovanni Luca Frassineti, Fabio Falcini, Rita Danesi

**Affiliations:** 1 Department of Medical Oncology, IRCCS Istituto Romagnolo per lo Studio dei Tumori “Dino Amadori” – IRST, 47014 Meldola, Italy; 2 Biosciences Laboratory, IRCCS Istituto Romagnolo per lo Studio dei Tumori “Dino Amadori” – IRST, 47014 Meldola, Italy; 3 Department of Medical Oncology, Degli Infermi Hospital, 47923 Rimini, Italy; 4 Romagna Cancer Registry, IRCCS Istituto Romagnolo per lo Studio dei Tumori “Dino Amadori” – IRST, 47014 Meldola, Italy; 5 Medical Oncology Unit, Department of Oncology AUSL Romagna, Degli Infermi Hospital, Rimini, Italy; 6 Oncology Unit, Ravenna Hospital, AUSL Romagna, Ravenna, Italy; 7 Scientific Directorate, IRCCS Istituto Romagnolo per lo Studio dei Tumori “Dino Amadori” – IRST, 47014 Meldola, Italy

**Keywords:** Pancreatic cancer, DNA damage repair, Gene panel, Cancer susceptibility, Targeted therapy

## Abstract

**Background:**

Pancreatic cancer (PC) is a major cause of cancer death. In an effort to improve treatment strategies and outcomes, DNA damage repair (DDR) pathways have been introduced as a new target in PC and in other cancers, through the exploitation of synthetic lethality. Furthermore, genes involved in DDR are among the major determinants of cancer susceptibility. In addition to the well-known *BRCA1* and *BRCA2* genes, a plethora of other targets in the same pathways are now emerging.

**Methods:**

We analyzed samples from 60 patients, affected by PC and already tested for *BRCA*, using a panel with 24 other cancer susceptibility genes.

**Results:**

We detected 8 pathogenic or likely pathogenic mutations (13.3% of samples analyzed), 4 of which were found in non-*BRCA* genes (2 in *ATM*, 1 each in *PALB2* and *RAD50*). Furthermore, 4 pathogenic or likely pathogenic mutations were found in patients without a personal or familial history of cancer.

**Conclusions:**

Our results suggest that genetic testing with a comprehensive gene panel should be perfomed in all patients with PC, in order to allow screening for PC and other gene-related cancers in all at risk family members and to assess patients’ eligibility for emerging therapeutic options.

## Background

Pancreatic cancer (PC) is the seventh leading cause of cancer death in the world [1], with a 5-year survival rate of about 9% [2]. Surgery is the only curative treatment, but no more than 20% of patients are eligible for resection [3], since the majority of cases are diagnosed at a late stage and are only amenable to systemic therapy. Despite recent advancements with chemotherapy combination regimens that resulted in increased survival [4, 5], the identification of new targets is critical to improve the efficacy of systemic therapy. Increasing attention is been paid to DNA damage repair (DDR) pathways in PC and in other diseases. Indeed, genomic instability and mutations are among the hallmarks of cancer [6]; genomic instability derives not only from an accumulation of mutations and other genetic alterations (e.g. induced by mutagen chemical compounds, ionizing or ultraviolet radiation) exacerbated by the replication stress in highly proliferating cells, but also from the impairment in repair pathways. These are based on a network of highly coordinated proteins that sense, signal and repair DNA damage, and coordinate this process with cell cycle progression [7]. Among the various mechanisms involved, the homologous recombination (HR) repair is critical for DNA double-strand breaks. The pathogenetic role of mutations in *BRCA1* and *BRCA2*, two key components of HR mechanism, has been largely established in several cancers such as breast, ovarian, prostate and PC [8], and *BRCA1/2* germline mutations are among the most common causes of inherited cancer susceptibility. Within this context, about 10% of PC cases have been linked to a familial predisposition [3], and *BRCA1* and *BRCA2* are among the most frequently mutated genes in familial PC [9]. Nevertheless, the majority of PC patients with *BRCA* mutations have no familial history, and germline *BRCA1* and *BRCA2* mutations are found in about 1 and 3.6% of patients, respectively, even without selection for familial history [10]. Moreover, alterations in DDR pathways play a role not only in inherited susceptibility to PC, but also in treatment of the disease, mainly through the exploitation of the so-called synthetic lethality, i.e. cell death resulting from simultaneous perturbation of the activity of two genes [11]. A common attempt to exploit this mechanism is a pharmacological intervention causing a DNA damage in a cell that is already deficient in a DDR pathway, e.g. using a platinum compound or a poly (adenosine diphosphate–ribose) polymerase inhibitor (PARPi) in *BRCA*-mutant cells. Indeed, *BRCA* mutations confer sensitivity to platinum-containing regimens in PC [12, 13]; furthermore, it has been recently established the role of the PARPi olaparib in maintenance therapy of *BRCA*-mutant PC after platinum-containing first-line treatment [14]. Studies in cancers other than PC (i.e. ovarian and prostate) have shown that *BRCA1* and *BRCA2* are not the only genes whose alteration is essential in this context: indeed, the wider concept of HR deficiency, including other genes such as *ATM* or *PALB2*, is implicated in the exploitation of synthetic lethality [15–20]. Furthermore, new treatment options that take advantage of this mechanism are emerging in addition to PARPi. Given the above premises, we decided to examine a series of samples from 60 consecutive cases of PC (from February 2019 to September 2020) analyzed for *BRCA1/2* status, and to broaden the analysis by including 24 other cancer susceptibility genes (*ABRAXAS1*, *ATM*, *APC*, *BARD1*, *BRIP1*, *CDH1*, *CHEK2*, *EPCAM*, *MLH1*, *MRE11*, *MSH2*, *MSH6*, *MUTYH*, *NBN*, *PALB2*, *PIK3CA*, *PMS2*, *PTEN*, *RAD50*, *RAD51C*, *RAD51D*, *STK11*, *TP53*, *XRCC2*).

## Methods

### Patient population

From February 2019 to September 2020, peripheral blood samples from 60 patients affected by PC were analyzed for *BRCA* status at the Biosciences Laboratory of the IRCCS Istituto Romagnolo per lo Studio dei Tumori “Dino Amadori” - IRST (formerly Istituto Scientifico Romagnolo per lo Studio e la Cura dei Tumori - IRST - IRCCS). All patients had a histological or cytological diagnosis of PC. Patients had been referred for *BRCA* testing by Medical Oncology Units (IRST IRCCS and other hospitals in the AUSL Romagna network) or by the Genetics Unit of IRST IRCCS, where, based on personal and familial history, they had been referred for counseling. Familial history refers to first- and second-degree relatives. The study was approved by the institutional review board (Ethics Committee IRST IRCCS-AVR, 2207/2012) and conducted in accordance with the Declaration of Helsinki. Patients have signed informed consent before analysis.

### Sample collection, DNA extraction and next-generation sequencing analysis

Peripheral blood samples were collected and stored at − 80 °C. Genomic DNA was extracted from blood using the QIAamp DNA Mini Kit (Qiagen, Hilden, Germany) and quantified using Qubit fluorometer (Thermo Fisher Scientific, Waltham, MA, USA) with Qubit dsDNA BR Assay Kit. The Next-Generation Sequencing (NGS) analysis was performed using the enrichment protocol of SOPHiA Hereditary Cancer Solution™ (HCS) v1.1 by SOPHiA GENETICS (Saint Sulpice, Switzerland) which analyzes 26 cancer predisposition genes (*ABRAXAS1*, *APC*, *ATM*, *BARD1*, *BRCA1*, *BRCA2*, *BRIP1*, *CDH1*, *CHEK2*, *EPCAM*, *MLH1*, *MRE11*, *MSH2*, *MSH6*, *MUTYH*, *NBN*, *PALB2*, *PIK3CA*, *PMS2*, *PTEN*, *RAD50*, *RAD51C*, *RAD51D*, *STK11*, *TP53*, *XRCC2*) and the pseudogene *PMS2CL*. Sequencing libraries were created starting from 200 ng of genomic DNA, following the HCS enrichment protocol for simultaneous sequencing of 26 genes. The Multigene Panel Testing (MGP) targets a total of 105 kb of the human genome and their flanking regions (on average 25 bp upstream and downstream each exon). DNA sequencing was performed with the MiSeq® Reagent Kit v3 600 cycles (Illumina, San Diego, CA, USA) on a MiSeq® platform (Illumina, San Diego, CA, USA), configured 2 × 151 cycles, according to manufacturer’s instructions.

### Data analysis and variant filtering

Sequences were mapped to the human reference genome GRCh37/hg19. Data output files (FASTQ) were uploaded on the SOPHiA DDM® Platform v5.5.0 (SOPHiA GENETICS, Saint Sulpice, Switzerland) for analysis. Custom filters were created to improve variant annotation and interpretation according to the assay. These included: alternative variant frequency higher than 30% (for detecting germline variants), and a minimum read depth of 50x per variant. The identified genetic variants were divided into five classes according to the International Agency for Research on Cancer (IARC) recommendations [21]: Pathogenic (PV - class 5), Likely Pathogenic (LPV - class 4), Variant of Unknown Significance (VUS - class 3), Likely Benign (LBV - class 2) and Benign (BV - class 1). Additional categories according to ClinVar interpretation including NA (Not Available) or Other, Risk Factor, Drug Response, Protective and Conflicting Interpretation, were merged with VUS. Variants automatically annotated by the platform were manually checked on the main human genomic databases. Variant classification was performed using the main mutation databases: BRCA Share™ (formerly Universal Mutation Database) [22], Leiden Open Variation Database (LOVD) [23], BRCA Exchange [24], ClinVar [25], dbSNP [26], HCI Cancer Susceptibility Genes Prior Probabilities of Pathogenicity [27], Varsome [28], and were categorized according to the available clinical interpretation [29]. All variants classified as PV/LPV were validated and confirmed through a second NGS-based analysis. Variants not included in any of these databases were classified according to the guidelines of the American College of Medical Genetics and Genomics (ACMG) [30]. This classification is based on variant characteristics: variants producing premature stop codons or gross deletions were considered pathogenic (PV-class 5) or likely-pathogenic (LPV-class 4).

## Results

### Patient population

From February 2019 to September 2020, samples from 60 patients with PC (60% male, 40% female) were analyzed (Table 1). All patients were Caucasian. Median age was 62 at diagnosis and 64 at testing. Personal history details were available for 49 patients (81.7%): 10 patients had a previous cancer diagnosis (5 breast, 2 colon, 1 prostate, 1 thyroid, 1 kidney, 1 non-Hodgkin lymphoma), while 39 had no previous history of cancer. Of the 32 patients (53.3%) with an available comprehensive family history, 23 reported a familial history of cancer in first- or second-degree relatives: 4 pancreas, 12 breast, 1 ovarian, 16 had at least a relative with another tumour (6 cases of stomach cancer, 5 colon, 5 lung, 4 prostate, 2 uterus, 2 urothelial tract, 1 kidney, 1 esophagus, 1 head and neck, 1 brain).

**Table 1:** Patient population characteristics

	n	%
**Patients**	60	100
Male	36	60
Female	24	40
**Age at diagnosis (years)**		
Median	62	
Range	43–81	
**Age at testing (years)**		
Median	64	
Range	43–81	
**Personal history**		
Available	49	81.7
Other cancer^a^	10	20.4
Breast	5^b^	
Ovarian	0	
Other	6	
No other cancer	39	79.6
Not available	11	18.3
**Family history**^c^		
Available	32	53.3
Cancer^a^	23	71.9
Pancreatic	4	
Breast	12	
Ovarian	1	
Other	16	
No cancer	9	28.1
Not available	28	46.7

^a^ some patients have history of ≥2 cancers; ^b^ 5 + 1 in situ; ^c^ first-grade and second-grade relatives

### Genetic variants

**Fig. 1:**
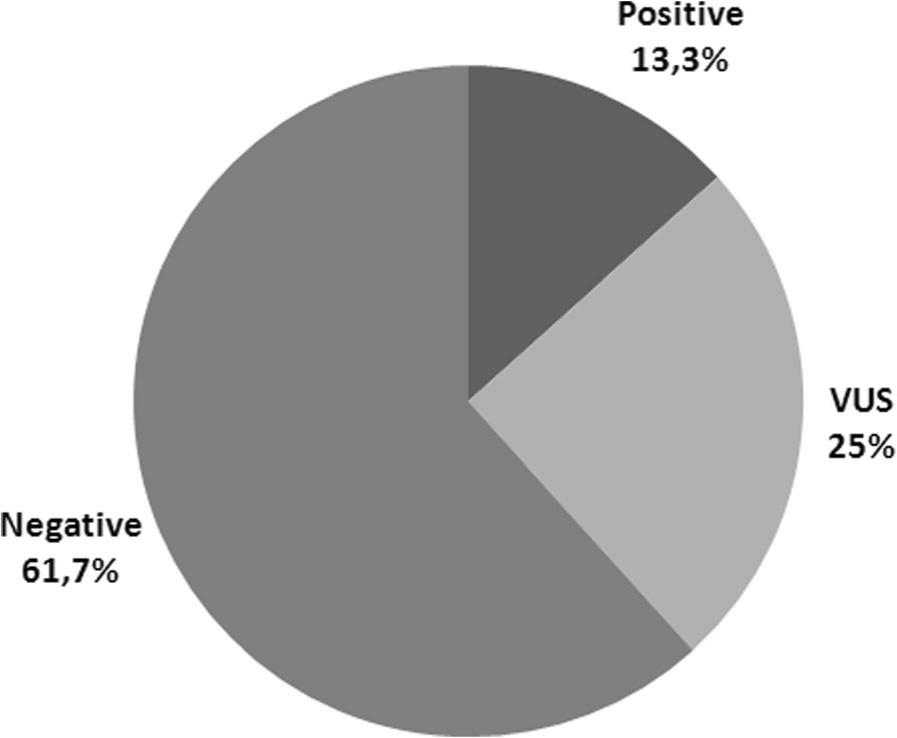
Percentage of patients with pathogenic or likely pathogenic mutation (positive), variants of uncertain significance (VUS) or no mutations (negative)

**Fig. 2:**
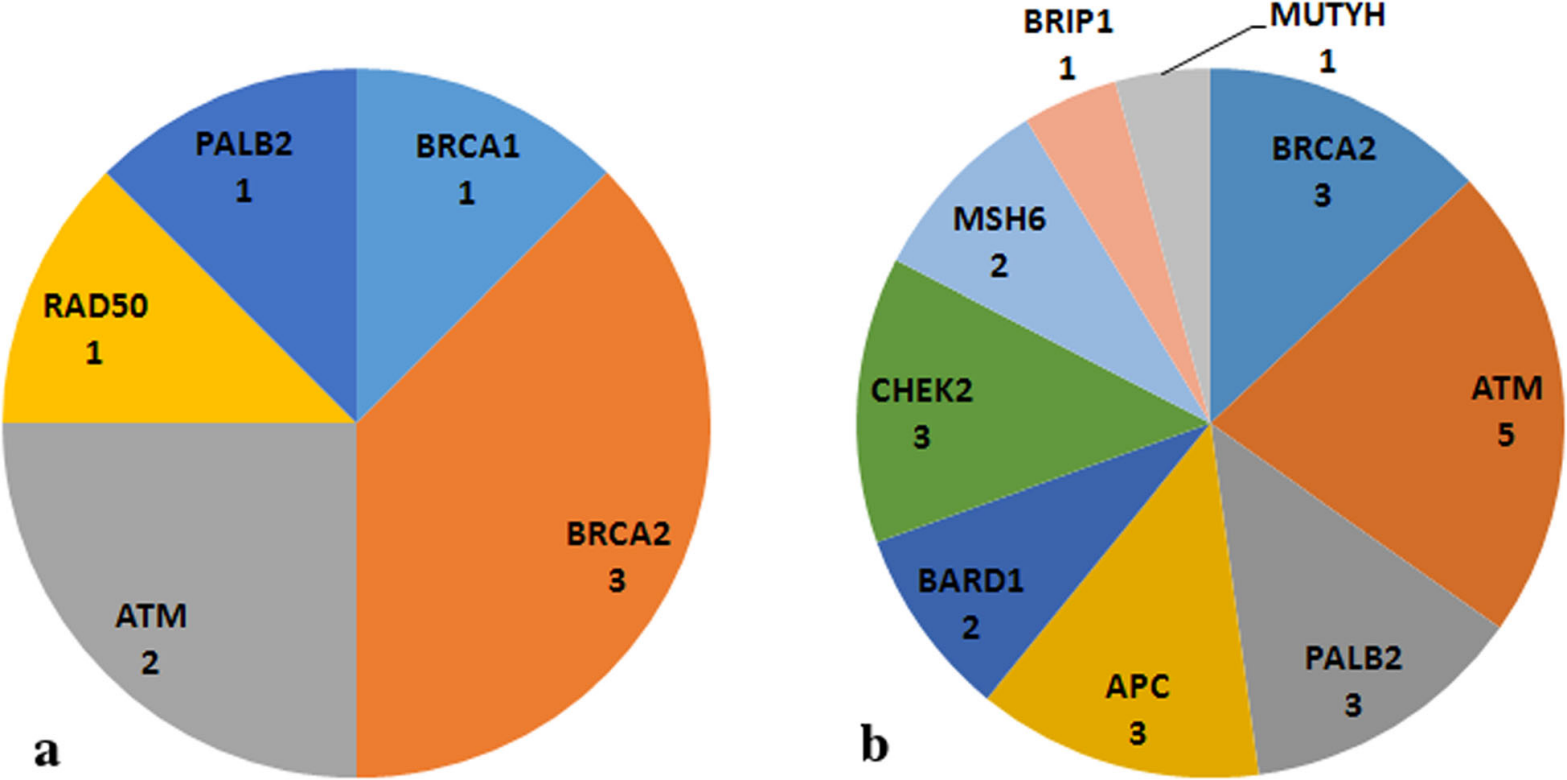
**a** Gene distribution of pathogenic and likely pathogenic mutations identified. **b** Gene distribution of variants of uncertain significance identified

**Fig. 3:**
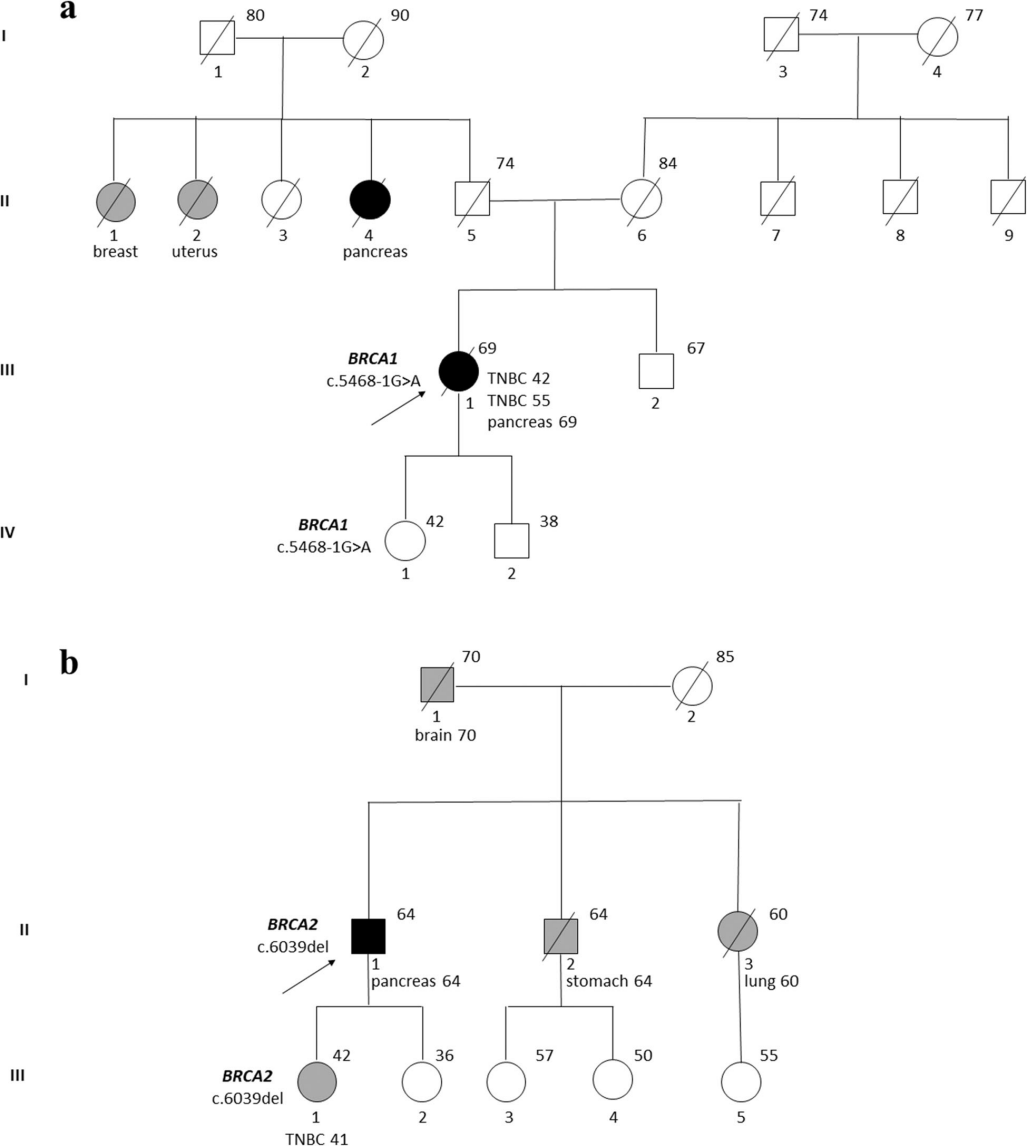
Pedigrees of a female patient with *BRCA1* mutation c.5468-1G > A (**a**) and a male patient with *BRCA2* mutation c.6039del (**b**) The probands are indicated by arrowheads. Symbols: square, male; circle, female; black, affected by PC; gray, affected by cancer other than PC; slashed symbol, deceased. Number above the symbols indicate age at death or last follow-up. Cancer type and age of onset are indicated below the symbols. TNBC, triple-negative breast cancer

PVs or LPVs were found in 8 out of 60 patients analyzed (13.3%). VUS were reported in 15 other patients (25%), while in 37 patients (61.7%) no variants were found (Fig. 1). Notably, one patient had 1 PV (in *BRCA2*) and 2 VUS (in *ATM* and *APC*); 5 other patients had 2 VUS in 2 different genes; one patient had 2 VUS in the same gene (*ATM*). Among the PVs and LPVs, 3 were found in *BRCA2*, 2 in *ATM*, 1 each in *BRCA1*, *PALB2* and *RAD50* (Fig. 2a and Table 2). None of the identified PVs or LPVs were found in more than one patient. Five single nucleotide variants, 2 deletions and 1 insertion have been found. Among the 23 VUS reported, 5 were detected in *ATM*, 3 each in *BRCA2*, *APC*, *CHEK2* and *PALB2*, 2 each in *BARD1* and *MSH6*, 1 each in *BRIP1* and *MUTYH* (Fig. 2b and Table 3). Only one mutation (c.2870A > G in *APC*) was found more than once (2 patients). Nineteen missense mutations, 2 copy number variations and 2 intronic variants were found. Of the 8 patients with a PV or LPV, only one had a previous history of cancer: a *BRCA1* mutation carrier, diagnosed with PC at the age of 69, had two triple-negative breast cancers (TNBCs), at 42 and 55 years. Four of the 8 patients had a familial history of cancer (none for PC; Table 2). Among the 15 patients with VUS, 5 had a previous personal history of cancer, and 5 had a familial history, of whom only one for PC (Table 3). The pedigrees of two patients harbouring a pathogenic *BRCA* mutation are shown in Fig. 3: a female patient with a *BRCA1* mutation (c.5468-1G > A) and a male patient with a *BRCA2* mutation (c.6039del). The former, who was diagnosed with PC at the age of 69, had a history of 2 TNBCs: the first when she was 42 and the second (contralateral) when she was 55. Her family history included one case of PC, one breast cancer and one endometrial cancer; her daughter, unaffected, carries the same *BRCA1* mutation. In the second case, the patient was diagnosed with PC at the age of 63, and had no previous personal history of cancer. Of note, his daughter, carrying the same *BRCA2* mutation, was diagnosed with TNBC when she was 41, and his family history included also one case of gastric cancer, one lung cancer and one brain cancer.

**Table 2:** List of pathogenic and likely pathogenic variants identified

Gene Transcript	cDNA change	Protein change	Variant Type	Consequence	IARC	Sex	Age at diagnosis	Personal history (age)	Familial history (cases)
*BRCA1* NM_007294	c.5468-1G > A	p.(?)	SNV	Splicing	C5	F	69	breast (42, 55)	pancreas (1), breast (1), uterus (1)
*BRCA2* NM_000059	c.6039del	p.(Val2014TyrfsTer26)	del	Frameshift	C5	M	63	none	breast (1), stomach (1), lung (1), brain (1)
*BRCA2* NM_000059	c.8364G > A	c.8364G > A	SNV	Missense	C5	M	72	none	NA
*BRCA2* NM_000059	c.1532_133insT	(p.Pro512ThrfsTer2)	ins	Frameshift	C5	M	61	none	lung (1), prostate (1)
*ATM* NM_000051	c.3275C > A	p.(Ser1092*)	SNV	Nonsense	C5	M	44	NA	NA
*ATM* NM_000051	c.4236 + 2 T > A	p.(?)	SNV	Splicing	C4	F	56	none	none
*PALB2* NM_024675	c.2167_2168del	p.(Met723Valfs*21)	del	Frameshift	C5	F	44	none	breast (1), colon (1), head and neck (1)
*RAD50* NM_005732	c.1636-1G > A	p.?	SNV	Splicing	C4	M	53	none	none

*M* Male, *F* Female, *IARC* International Agency for Research on Cancer classification (C5: pathogenic; C4: likely pathogenic), *SNV* Single Nucleotide Variation, *NA* Not available

**Table 3:** List of variants of uncertain significance identified

Gene Transcript	cDNA change	Protein change	Variant type	Consequence	Sex	Age at diagnosis	Personal history (age)	Familial history (cases)
*BRCA2* NM_000059	c.9613_9614delinsCT	p.(Ala3205Leu)	delins	Missense	F	62	none	none
*BRCA2* NM_000059	c.1705C > A	p.(Gln569Lys)	SNV	Missense	M	61	none	NA
*BRCA2* NM_000059	c.476 T > C	p.(Val159Ala)	SNV	Missense	M	62	kidney (62)	lung (1), kidney (1), stomach (1), esophagus (1)
*APC* NM_000038	c.1450G > C	p.(Glu484Gln)	SNV	Missense	M	63	none	breast (1), stomach (1), lung (1), brain (1)
*APC* NM_000038	c.2870A > G	p.(Lys957Arg)	SNV	Missense	M	61	none	none
*APC* NM_000038	c.2870A > G	p.(Lys957Arg)	SNV	Missense	F	64	breast (40), thyroid (53, 59)	colon (1), stomach (1)
*ATM* NM_000051	c.5975A > C	p.(Lys1992Thr)	SNV	Missense	M	63	none	breast (1), stomach (1), lung (1), brain (1)
*ATM* NM_000051	c.1464G > T	p.(Trp488Cys)	SNV	Missense	M	66	none	none
*ATM* NM_000051	c.8734A > G	p.(Arg2912Gly)	SNV	Missense	M	58	none	none
*ATM* NM_000051	c.8671 + 17A > G	p.(?)	SNV	Intronic	M	71	NA	NA
*ATM* NM_000051	c.2376 + 16del	p.(?)	SNV	Intronic	M	71	NA	NA
*BARD1* NM_000465	c.2251C > T	p.(Arg751Trp)	SNV	Missense	M	66	none	none
*BARD1* NM_000465	c.2027A > G	p.(Tyr676Cys)	SNV	Missense	M	61	none	NA
*BRIP1* NM_032043	c.845C > G	p.(Thr282Ser)	SNV	Missense	M	45	none	none
*CHEK2* NM_007194	c.793_846del	p. (?)	CNVs	Large deletion	F	79	colon (68), breast (71)	breast (1)
*CHEK2* NM_007194	c.500G > A	P. (Gly167Glu)	SNV	Missense	M	61	none	none
*CHEK2* NM_007194	c.118A > G	p.(Ser40Gly)	SNV	Missense	F	75	none	NA
*MSH6* NM_000179	c.1660C > T	p. (Arg554Cys)	SNV	Missense	F	62	none	none
*MSH6* NM_000179	c.3515G > T	p. (Arg1172Ile)	SNV	Missense	F	67	breast (40, 47, 61)	NA
*MUTYH* NM_001128425	c.1483C > T	p.(Arg495Cys)	SNV	Missense	M	60	prostate (49)	breast (1)
*PALB2* NM_024675	c.109_211dup	p. (?)	CNVs	Large duplication	M	62	kidney (62)	lung (1), kidney (1), stomach (1), esophagus (1)
*PALB2* NM_024675	c.2453 T > C	p.(Phe818Ser)	SNV	Missense	M	60	prostate (49)	breast (1)
*PALB2* NM_024675	c. 3296C > T	p. Thr1099Met	SNV	Missense	M	58	none	pancreas (1), prostate (1)

*M* Male, *F* Female, *SNV* Single Nucleotide Variation, *CNVs* Copy Number Variations, *NA* Not available

### Analysis by medical history

We then analyzed the incidence of mutations based on medical history. Of 10 patients with a personal history of cancer, one had a PV, 5 VUS and 4 no mutations; in 50 patients with negative or unknown history, 7 had PVs or LPVs, 10 VUS and 33 no mutations. Of 23 patients with a familial history of cancer, 4 had a PV or LPV, 5 VUS and 14 no mutations; in 37 patients with negative or unknown familial history, 4 had a PV or LPV, 10 VUS and 23 no mutations (Table 4).

**Table 4:** Analysis by medical history. Mutations are reported according to personal and familial history for cancer

	Mutations		
	C4/C5	VUS	no
**Personal history**			
**Yes**	1	5	4
**No/unknown**	7	10	33
**Familial history**			
**Yes**	4	5	14
**No/unknown**	4	10	23

C4/C5 Likely pathogenic (C4) or pathogenic (C5) according to the IARC classification, VUS Variants of uncertain significance

## Discussion

The rate of *BRCA* mutations in our case series (3 *BRCA2*, 1 *BRCA1*: total 6.67%) as well as the ratio between the two genes are consistent with other reports [10]. The number of patients with a familial history of PC (4/32, 12.5%) is also consistent with other reports [10]. While the rate of a positive history for breast cancer was as expected (5/49 for personal history, 12/32 for familial history), a remarkable finding in our case series is the rarity of ovarian cancer, a tumor often related to *BRCA* mutations: only one case reported a familial history of ovarian cancer, whereas none of the patients had had a previous diagnosis of this disease. Similar considerations emerge from the two pedigrees shown in Fig. 3: in both cases there was a history of TNBC, a tumor often associated with a *BRCA* mutation, especially at a younger age (the same patient carrying a *BRCA1* mutation, the patient’s daughter for *BRCA2* mutation); the *BRCA1* mutation carrier had an aunt with PC, and none of the two patients had relatives with a history of ovarian cancer.

In addition to *BRCA*, we found pathogenic or likely pathogenic mutations in genes involved in HR in another 4 patients (2 *ATM*, 1 *PALB2*, 1 *RAD50*), making a total of 13.3% of patients carrying a pathogenic mutation, that is still consistent with other reports [31]. Whilst the role of *PALB2* and *ATM* is fairly well established in PC [9, 32–34], *RAD50* is not among the genes generally associated with this tumour. We detected a likely pathogenic variant (c.1636-1G > A) in *RAD50*, in a patient whose family history was unremarkable. The RAD50 protein is a member of the structural maintenance of chromosome protein family, and is part of a complex, together with MRE11 and NBS1, involved in DNA double-strand break repair [35]. *RAD50* mutations have previously been reported in PC, both at germline and somatic level, suggesting a possible role of *RAD50* as a PC predisposition gene [36, 37]. At the same time, RAD50 protein has been found upregulated in serum of patients affected by PC, and a possible negative feedback mechanism has been proposed [38].

A potential limitation of our study is the lack of *CDKN2A* in the gene panel. This gene is frequently mutated in familial PC and is associated with the familial atypical mole and melanoma syndrome [9]. Indeed, the kit we used, SOPHiA HCS, is more focused on hereditary breast and ovarian cancer, Lynch syndrome and intestinal polyposis syndromes, that do not include *CDKN2A* among the most relevant genes. Nevertheless, *CDKN2A* is not directly involved in DDR; rather, its main products, p16INK4a and p14ARF, are tumor suppressors involved in cell cycle regulation. Indeed, p16INK4a interacts with CDK4 and CDK6, inhibiting their interaction with cyclin D and pRb phosphorylation, thus preventing transition from G1 to S phase; p14ARF induces cell cycle arrest by activating p53 through the inhibition of its negative regulator MDM2 [39]. Therefore, given that our aim was to investigate DDR-related genes rather than genes merely involved in cancer susceptibility syndromes, we considered the SOPHiA HCS as a valid tool for this task.

A noteworthy consideration comes from the analysis based on medical history (Table 4). Had we limited our analysis to patients with a personal or familial history of cancer, 4 pathogenic or likely pathogenic mutations would have been reported, but 4 more mutations would have been missed (in addition to 13 VUS). Furthermore, as seen in our series, familial history is often incomplete in clinical records, for several reasons such as i) difficulty in its retrieval and ii) genetic testing often performed in different centers with respect to the oncology clinics where patients undergo treatment. This bias may result in the mistake of considering a lacking history as a negative one, thus excluding patients with a potential positive history from genetic testing. This raises attention on the likely underestimation of the ratio of mutations in *BRCA* and other genes if patients’ selection for testing is based solely on personal and familial history.

Another important consideration is our extension of testing to other DDR-related genes in addition to *BRCA*: in our case series, we detected 4 *BRCA* mutations (3 *BRCA2*, 1 *BRCA1*) and 4 mutations in other genes (2 *ATM*, 1 *PALB2*, 1 *RAD50*). Indeed, the role of genes other than *BRCA* in cancer susceptibility inheritance is well established, and PC is part of the clinical spectrum in several syndromes (e.g. Lynch syndrome from mismatch repair (MMR) gene mutations, Peutz-Jeghers syndrome from *STK11* mutations) [9]. Moreover, our knowledge about other cancers (e.g., ovary, prostate) underscores the possible therapeutic implications of a broader range of DDR gene mutations [15–20], and this concept has recently been extended to PC [40, 41]. This highlights the need for genetic screening beyond *BRCA*: in our opinion it is mandatory to take advantage of a gene panel that cannot exclude essential genes such as *ATM*, *PALB2*, *RAD50*, *STK11* and MMR genes [9, 31, 40]. The technical advancements and the more affordable costs resulting from the application of high-throughput methods (NGS) make such an approach feasible. In the near future, it is likely that even more alterations will be investigated, since about 450 proteins are involved in DDR [7], many of which are druggable targets currently under investigation.

In PC DDR alterations are common both at germinal and somatic level [41]. A comprehensive genomic analysis (whole-genome sequencing and copy number variation) of 100 cases of PC resulted in a classification into 4 subtypes according to chromosomal structural variation: stable, locally rearranged, scattered and unstable [42]. The unstable subtype, accounting for 14% of cases, exhibited a large number of structural variation events, and was associated with DDR defects (including, but not limited to, *BRCA1/2*, *PALB2*, *ATM*), along with platinum responsiveness [42]. Indeed, while in some cancers *BRCA* mutations appear to be biologically neutral, in PC they have a paramount phenotypic importance and, if present, they emerge as an indispensable founding event [43]. Based on previous considerations, we can speculate that this is also true for other DDR alterations.

Given the above premises, the identification of all mutation carriers is critical for both risk reduction and therapeutic strategy. With regard to risk reduction, the identification of all mutation carriers would allow for a tailored follow-up of patients (aimed at the early detection of secondary tumours) and would facilitate cascade testing and screening for PC and other gene-related cancers in all at risk family members. As for therapeutic strategy, knowledge of a *BRCA* or *PALB2* mutation would orient first-line treatment towards a platinum-containing regimen, given the known sensitivity of *BRCA*- and *PALB2*-mutated PC to platinum-based therapy [12, 13, 44]; furthermore, disease control after a platinum-containing regimen would enable patients to undergo maintenance with olaparib [14]. In addition to the opportunities coming from the increasing number of clinical trials focusing on tumours with DDR defects [41], knowledge of a mutation in this pathway would give patients a potential therapeutic option that would otherwise be lost if overly restrictive eligibility criteria (i.e. based on familial history) excluded such mutated cases from testing or if the analysis were limited to *BRCA1* and *BRCA2*. Indeed, up to 25% of PCs harbour actionable molecular alterations, the majority of which are in the DDR pathway [45]. Together with the first approval of a targeted treatment (olaparib in maintenance therapy of *BRCA*-mutated PC) [14], new approaches, including drug combinations, are being evaluated to increase the efficacy of available treatments, increase the number of eligible patients, and counteract resistance mechanisms. Many of these approaches aim to induce or maintain HR defectiveness, also by inhibiting targets in other pathways, such as PI3K, MEK, WEE1 [41].

## Conclusions

In conclusion, given the potential therapeutic and family prevention implications outlined above, we strongly endorse genetic testing for all patients with a confirmed diagnosis of PC, as already suggested by some international guidelines [46]. This would translate in a change of paradigm: while the first step for *BRCA* analysis used to be genetic counseling which, on the basis of family history, advised testing or not, the new approach would offer genetic testing as soon as received the diagnosis of PC (e.g. by the oncologist) and subsequent genetic counseling only in the event of positive (or uncertain) results, or for patients with a family history of cancer. Furthermore, our results also indicate that genetic testing should not solely be based on *BRCA1* and *BRCA2*, but rather on a comprehensive gene panel including at least *ATM*, *PALB2*, *RAD50*, *STK11* and MMR genes.

## Data Availability

The dataset generated and analysed during the current study is not publicly available due to privacy issues but is available, in anonymized form, upon reasonable request.

## Abbreviations

DDR: DNA damage repair
HR: Homologous recombination
IARC: International Agency for Research on Cancer
LPV: Likely pathogenic variant
MMR: Mismatch repair
NGS: Next-Generation Sequencing
PARPi: Poly (adenosine diphosphate–ribose) polymerase inhibitor
PC: Pancreatic cancer
PV: Pathogenic variant
TNBC: Triple-negative breast cancer
VUS: Variant of uncertain significance
